# N-terminal Serine Dephosphorylation Is Required for KCC3 Cotransporter Full Activation by Cell Swelling*

**DOI:** 10.1074/jbc.M113.475574

**Published:** 2013-09-16

**Authors:** Zesergio Melo, Paola de los Heros, Silvia Cruz-Rangel, Norma Vázquez, Norma A. Bobadilla, Herminia Pasantes-Morales, Dario R. Alessi, Adriana Mercado, Gerardo Gamba

**Affiliations:** From the ‡ Molecular Physiology Unit, Instituto de Investigaciones Biomédicas, Universidad Nacional Autónoma de México and Instituto Nacional de Ciencias Médicas y Nutrición Salvador Zubirán, Tlalpan, 14000 Mexico City, Mexico,; the § Medical Research Council Protein Phosphorylation and Ubiquitylation Unit, College of Life Sciences, University of Dundee, Dow Street, Dundee DD1 5EH, Scotland, United Kingdom,; the ¶ División de Neurociencias, Instituto de Fisiología Celular, Universidad Nacional Autónoma de México, 04510 Mexico City, Mexico, and; the ‖ Departamento de Nefrología, Instituto Nacional de Cardiología Ignacio Chávez, Tlalpan, 14080 Mexico City, Mexico

**Keywords:** Hypertension, Phosphorylation, Physiology, Potassium Transport, Signal Transduction

## Abstract

The K^+^:Cl^−^ cotransporter (KCC) activity is modulated by phosphorylation/dephosphorylation processes. In isotonic conditions, KCCs are inactive and phosphorylated, whereas hypotonicity promotes their dephosphorylation and activation. Two phosphorylation sites (Thr-991 and Thr-1048) in KCC3 have been found to be critical for its regulation. However, here we show that the double mutant KCC3-T991A/T1048A could be further activated by hypotonicity, suggesting that additional phosphorylation site(s) are involved. We observed that *in vitro* activated STE20/SPS1-related proline/alanine-rich kinase (SPAK) complexed to its regulatory MO25 subunit phosphorylated KCC3 at Ser-96 and that in *Xenopus laevis* oocytes Ser-96 of human KCC3 is phosphorylated in isotonic conditions and becomes dephosphorylated during incubation in hypotonicity, leading to a dramatic increase in KCC3 function. Additionally, WNK3, which inhibits the activity of KCC3, promoted phosphorylation of Ser-96 as well as Thr-991 and Thr-1048. These observations were corroborated in HEK293 cells stably transfected with WNK3. Mutation of Ser-96 alone (KCC3-S96A) had no effect on the activity of the cotransporter when compared with wild type KCC3. However, when compared with the double mutant KCC3-T991A/T1048A, the triple mutant KCC3-S96A/T991A/T1048A activity in isotonic conditions was significantly higher, and it was not further increased by hypotonicity or inhibited by WNK3. We conclude that serine residue 96 of human KCC3 is a third site that has to be dephosphorylated for full activation of the cotransporter during hypotonicity.

## Teaser

**Background:** KCC3 lacking the two known phosphorylation sites is still regulated by cell swelling and WNK3.

**Results:** Dephosphorylation of serine 96 is necessary for full activation of the cotransporter.

**Conclusion:** Serine 96 is a third phospho-site involved in KCC3 regulation.

**Significance:** The finding of new phosphorylation sites sheds light on an increasingly complex regulation of K^+^:Cl^−^ cotransporters.

## Introduction

The K^+^:Cl^−^ cotransporters (KCCs)^4^ constitute a branch of the electroneutral cation-coupled chloride cotransporter family SLC12 composed of four members: KCC1 to KCC4. Following the driving force imposed by the Na^+^:K^+^:ATPase, the KCCs translocate ions from the inside to the outside of the cell participating in fundamental physiological processes such as regulatory volume decrease, trans-epithelial ion transport, and reduction of intracellular chloride concentration. Erythroid function and differentiation, cancer cell growth and invasiveness, arterial blood pressure regulation, and neuronal excitability are some of the important roles of these membrane proteins (1–3). Inactivating mutations of the *Slc12A6* gene encoding KCC3 are the cause of a complex neurological disease known as agenesis of the corpus callosum with peripheral neuropathy, also referred as Andermann syndrome (Online Mendelian Inheritance in Man (OMIM) 218000) (4–6). Moreover, in addition to reproducing this disease, the KCC3 knock-out mouse model also develops arterial hypertension (7).

The other branch of the SLC12 family is composed of the Na^+^-coupled chloride cotransporters, generally called N(K)CCs, that following the driving force imposed by the Na^+^:K^+^:ATPase translocate ions from the outside to the inside of the cell, thus having similar important roles as the KCCs in many physiological aspects, but in the opposite direction (1). As expected, NKCC and KCC activity is reciprocally regulated. Phosphorylation promoted by cell shrinkage, intracellular chloride depletion, or protein phosphatase inhibitors increases NKCCs and reduces KCC activity, whereas, dephosphorylation associated with cell swelling, intracellular chloride accumulation, or protein phosphatases decreases NKCC function and triggers KCCs (8).

For NKCCs, a cluster of 3–5 highly conserved threonine/serine residues, localized in the N-terminal domain, has been identified as a key regulator of the cotransporter activity (9, 10). Phosphorylation of these sites by kinases SPAK and oxidative stress-responsive kinase 1 (OSR1) in response to osmotic stress increases their activity. In contrast, for the KCC branch, two threonine residues at the C-terminal domain have been shown to be critical for KCC3 activity regulation (11). Phosphorylation of these sites renders the cotransporter inactive, and it becomes active after dephosphorylation. Here we show, however, that the N-terminal domain serine 96 of human KCC3a fulfills the characteristics for a third phosphorylation site involved in regulation of this cotransporter.

## EXPERIMENTAL PROCEDURES

### 

#### 

##### Mutagenesis and Constructs

Mutant constructs were prepared with the QuikChange mutagenesis system (Stratagene) and custom-made primers (Sigma). All mutations were confirmed by sequencing and subcloned back into the appropriate expression constructs.

##### Functional Expression of KCCs

We assessed the activity of wild type or mutant KCC3 or KCC4 using the heterologous expression system of *Xenopus laevis* oocytes as described previously (12–15). In brief, mature oocytes were injected with wild type or mutant KCC3 or KCC4 cRNA at 10 ng/oocyte, and 3 days later, the activity of the cotransporter was determined by assessing the Cl^−^-dependent, ^86^Rb^+^ uptake in isotonic or hypotonic conditions. cRNA for injection was transcribed *in vitro* from KCCs cDNA linearized at the 3′ using the T7 RNA polymerase mMESSAGE kit (Ambion). All experimental data are based on a minimum of three different experiments. Our institutional committee on animal research approved the use of *X. laevis* frogs.

##### Expression and Purification of GST-tagged SPAK DA, KCC3a(1–175), and NKCC2(1–174) in Escherichia coli

All pGEX-6P-1 constructs were transformed into BL21 *E. coli* cells, and 1-liter cultures were grown at 37 °C in LB medium (100 μg/ml ampicillin) until the absorbance at 600 nm was 0.8. Isopropyl β-d-thiogalactopyranoside (30 μm) was then added, and the cultures were grown for further 16 h at 26 °C. Cells were isolated by centrifugation, resuspended in 40 ml of ice-cold lysis buffer, and sonicated (Branson Digital Sonifier; ten 15-s pulses with a setting of 45% amplitude) to fragment DNA. Lysates were centrifuged at 4 °C for 15 min at 26,000 × *g*. The GST fusion proteins were affinity-purified on 0.5 ml of glutathione-Sepharose and eluted in buffer A containing 0.27 m sucrose and 20 mm glutathione.

##### KCC3a Phosphorylation Site Mapping by SPAK

GST-KCC3a(1–175) and GST-NKCC2(1–174) (10 μg) purified from *E. coli* were incubated with active SPAK (Carna Biosciences STLK3 (STK39), product number: 07-130) (1 μg) or kinase-inactive SPAK GST-SPAK(D212A) (1 μg) also purified from *E. coli* at 30 °C for 60 min in buffer A containing 10 mm MgCl_2_, 0.1 mm [γ-^32^P]ATP (∼15,000 cpm/pmol) in a total reaction volume of 25 μl. The reaction was terminated by the addition of LDS sample buffer. Dithiothreitol (DTT) was added to a final concentration of 10 mm, and the samples were heated at 95 °C for 4 min and cooled for 20 min at room temperature. Iodoacetamide was then added to a final concentration of 50 mm, and the samples were left in the dark for 30 min at room temperature to alkylate cysteine residues. The samples were subjected to electrophoresis on a Bis-Tris 10% polyacrylamide gel, which was stained with colloidal blue and then autoradiographed. The phosphorylated GST-KCC3a(1–175) and GST-NKCC2(1–174) bands were excised, cut into smaller pieces, and washed sequentially for 10 min on a vibrating platform with 1 ml of the following: water; 1:1 (v/v) mixture of water and acetonitrile; 0.1 m ammonium bicarbonate; 1:1 mixture of 0.1 m ammonium bicarbonate and acetonitrile; and finally acetonitrile. The gel pieces were dried and incubated for 16 h at 30 °C in 25 mm triethylammonium bicarbonate containing 5 μg/ml trypsin as described previously (10, 16). Following tryptic digestion, >95% of the ^32^P radioactivity incorporated in the gel bands was recovered, and the samples were chromatographed on a Vydac 218TP5215 C_18_ column (Separations Group, Hesperia, CA) equilibrated in 0.1% trifluoroacetic acid in water. The column was developed with a linear acetonitrile gradient (see Fig. 2*B*, *diagonal line*) at a flow rate of 0.2 ml/min, and fractions of 0.1 ml were collected.

##### Phospho-peptide Sequence Analysis

Isolated phospho-peptides were analyzed by LC-MSMS on a Thermo Scientific Orbitrap Classic. Raw files were processed using the Raw2msm application, and the resultant data were searched against an in-house database using Mascot search engine software. The site of phosphorylation of all the ^32^P-labeled peptides was determined by solid-phase Edman degradation on an Applied Biosystems 494C sequencer of the peptide coupled to Sequelon-AA membrane (Applied Biosystems) as described previously (17).

##### Antibodies

The following antibodies were raised in sheep and affinity-purified on the appropriate antigen: KCC3 total (residues 1–175 of human KCC3a), KCC3 phospho-Ser-96 (residues 80–94 or 89–103 of human KCC3a), KCC3 phospho-Thr-982 (residues 975–989 or 984–998 of human KCC3a), and KCC3 phospho-Thr-1039 (residues 1032–1046 or 1041–1055 of human KCC3a). Secondary antibodies coupled to horseradish peroxidase used for immunoblotting were obtained from Pierce. Specificity of phospho-antibodies was assessed using protein extracts from HEK293 transfected with KCC3 cDNA.

##### Stable and Transient Cell Lines and Cell Culture

A stably transfected HEK293 cell line developed for a previous study (19) that had pCDNA3.1 expression vector with the open reading frame of full-length human with no lysine kinase 3 (WNK3) was used to transiently transfect full-length human KCC3a. To generate the transient cell lines, pre-cultures of HEK293-WNK3 underwent passages every 2–3 days to keep the cells in their exponential growth phase. 2 h before transfection, the cell suspension was centrifuged (300 × *g* for 5 min), resuspended in fresh medium at 5 × 10^6^ cell/ml, and plated on 60-mm diameter dishes (60% confluence). Then, cells were transfected at 37 °C with the plasmid DNA or empty vector using the calcium phosphate method. Briefly, 5 μg of the full-length KCC3a was suspended in CaCl_2_ solution at a final concentration of 0.3 m and mixed with 150 μl of 2× HEPES-buffered saline (in mm: 50 HEPES, 280 NaCl, and 1.5 Na_2_HPO_4_, pH 7.05) dropwise while mixing gently. The mixture was left at room temperature for 30 min and then incubated with the cells for 6 h in 37 °C. Cells were then washed two times with 1× phosphate-buffered saline (PBS) and placed in Dulbecco's modified Eagle's medium (DMEM) supplemented with 10% FBS, 50 units/ml penicillin, 50 μg/ml streptomycin, and 1 mg/ml G418 in a humidified atmosphere containing 5% CO_2_ and 95% air at 37 °C. 48 h after transfection, cells were exposed to isotonic (in mm: 160 NaCl, 5 KCl, 1.17 MgSO_4_, 1 CaCl_2_, 5 glucose, and 10 HEPES, pH 7.4) or hypotonic 30% solutions (obtained by reduction of NaCl concentration) for 30 min. After treatment, the cells were harvested in ice-cold lysis buffer (in mm: 20 Tris/HCl, pH 7.4, 1 EDTA, 50 NaCl, 1 EGTA, 0.5 Na_3_VO_4_, 1 2β-glycerophosphate, and 1% Triton X-100), incubated for 10 min and then scraped. Cells homogenates were sonicated and centrifuged (11,000 × *g* for 20 min), and finally the supernatants were collected.

##### Immunoblotting

Groups of 10–20 oocytes were injected with cRNA from various constructs, transferred to Eppendorf tubes, and homogenized at 4 °C in lysis buffer (in mm 10 Tris-HCl, 150 NaCl, 1 EDTA, 1% Triton (10 μl/oocyte)) supplemented with protease and phosphatase inhibitor mixture (Roche Diagnostics). After clearing the lysate by centrifugation at 10,000 × *g* at 4 °C for 30 min, total protein was collected from the supernatant. Western blotting was carried out using previously characterized anti-KCC3 rabbit polyclonal antibodies (14) and the specific phospho-antibodies mentioned above. Total protein equivalent to one oocyte was separated in 7.5% polyacrylamide gels, transferred onto membranes (PVDF; Amersham Biosciences), blocked, and incubated overnight with the specific antibody at 3 μg/ml in TBST, 5% milk at 4 °C. After several washes with TBST (Tris-buffered saline/Tween 20, in mm: 100 Tris/HCl, 150 NaCl, and 0.1% Tween 20, pH 7.5), membranes were incubated with horseradish peroxidase-conjugated secondary antibody in blocking solution (1:7000, GE Healthcare Bioscience) and visualized by chemiluminescence (Amersham Biosciences ECL-Plus, GE Healthcare).

Cells cultured on 60-mm dishes were washed and then scraped into lysis buffer. Cell homogenates were sonicated and clarified by centrifugation (11,000 × *g* for 5 min), and protein concentration was determined by the Bradford method. Then, 25 μg of protein was separated by SDS-PAGE (7.5% acrylamide gel) and transferred onto PVDF membranes (Bio-Rad). Membranes were blocked with TBST containing 5% (w/v) nonfat dried milk and incubated overnight at 4 °C, with rabbit primary antibodies anti-KCC3 (1:1000) (14), anti-KCC3 phospho-Ser-96, anti-KCC3 phospho-Thr-991 (3 μg/ml plus 2 μl/ml non-phospho-peptide), or anti-β-actin (Santa Cruz Biotechnology). After further washing, blots were incubated with HRP-conjugated goat anti-rabbit IgG antibody (1:5000; Zymed Laboratories Inc.), except for KCC3 phospho-Ser-96 and phospho-Thr-991 that were incubated with an anti-sheep secondary antibody for 1 h at room temperature. Chemiluminescent reaction was assayed using ECL®-Plus Western blot detection reagents (GE Healthcare) according to the manufacturer's recommendations, and bands were visualized with exposure to Kodak BioMax light films (Sigma).

##### Statistical Analysis

Statistical significance is defined as two-tailed *p* < 0.05, and the results are presented as mean ± S.E. The significance of the differences between two groups was tested by Student's *t* test, and the significance of the differences for three or more groups was tested by one-way analysis of variance with multiple comparisons using Bonferroni's correction.

## RESULTS

### 

#### 

##### Evidence for Additional Phosphorylation Sites for KCC3 Regulation

**FIGURE 1.**
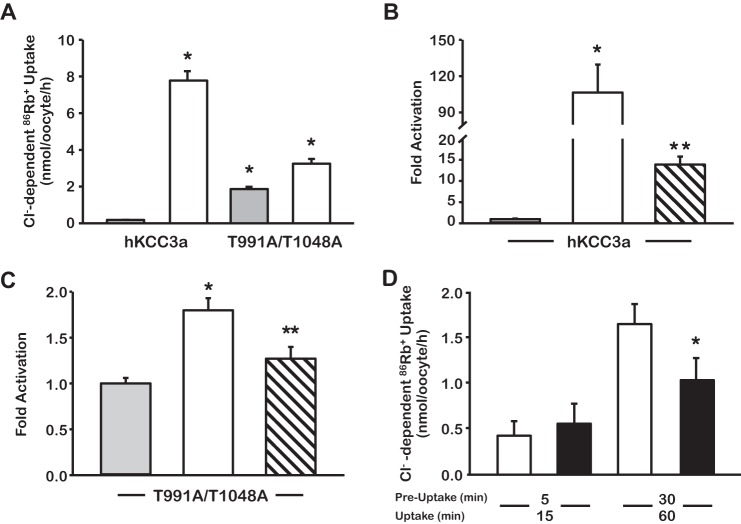
**Evidence for additional phosphorylation sites in KCC3**. *A*, functional expression assay shows the activity of wild type (*WT*) KCC3 and KCC3-T991A/T1048A under isotonic (*gray bars*) and hypotonic (*open bars*) conditions. *, *p* < 0.01 *versus* human KCC3a (*hKCC3a*) in isotonic condition. *B* and *C*, -fold activity of WT KCC3a (*B*) or KCC3a-T991A/T1048A (*C*) taking isotonic conditions as 100% (*gray bars*) and normalizing accordingly the effect of hypotonic conditions alone (*open bars*) or hypotonic conditions plus co-injection with WNK3 cRNA (*hatched bars*). *, *p* < 0.01 *versus* isotonic control. **, *p* < 0.05 *versus* hypotonicity without WNK3. *D*, time dependence of WT KCC3a activity (*open bars*) when compared with KCC3a-T991A/T1048A (*black bars*) in hypotonic conditions was measured after 5 or 30 min of pre-uptake incubation. *, *p* < 0.01 *versus* wild type.

The functional properties of wild type KCC3 (Fig. 1, *A* and *B*) were compared with those of the double mutant KCC3-T991A/T1048A (Fig. 1, *A* and *C*), in both isotonic and hypotonic conditions. As shown previously (14), wild type KCC3 was inactive in isotonic conditions (0.26 ± 0.053 nmol/oocyte/h) and incubation in hypotonicity dramatically increased its activity (7.83 ± 0.94 nmol/oocyte/h; *p* > 0.001). As reported by Rinehart *et al.* (11), we observed that ^86^Rb^+^ uptake in oocytes injected with the mutant KCC3-T991A/T1048A cRNA revealed a significant activity in isotonic conditions (2.0 ± 0.13 nmol/oocyte/h; *p* < 0.01 *versus* wild type in isotonicity). However, when oocytes were incubated in hypotonicity, a further increase in activity was observed (3.45 ± 0.40 nmol/oocyte/h; *p* < 0.0001 *versus* uptake in isotonicity). Additionally, as depicted in Fig. 1, *B* and *C*, and reported previously for wild type KCC3 (15, 18, 19), the activity of both wild type KCC3 and KCC3-T991A/T1048A in hypotonic conditions was significantly reduced by co-expression with the WNK3 (2.98 ± 0.37 and 2.87 ± 0.046 nmol/oocyte/h, respectively, *p* < 0.05 *versus* the absence of WNK3). These observations suggested that there might be an additional phosphorylation site(s) that has to be dephosphorylated to achieve full activation of the cotransporter. This proposal differs from that of Rinehart *et al.* (11) where dephosphorylation of threonines 991 and 1048 was suggested to be sufficient for full activation of the cotransporter. In their study, however, wild type KCC3 was incubated in hypotonic conditions for a total of 8 min, whereas it has been reported that KCCs require longer incubation times to reach full activation (20–22). Thus, it is possible that the activity of the double mutant KCC3-T991A/T1048A in isotonicity was higher than wild type KCC3 in hypotonicity because this latter was not yet fully active at the time of the uptake assay. To assess this possibility, we measured the activity of wild type KCC3 and the double mutant KCC3-T991A/T1048A after 5 or 30 min of pre-uptake incubation followed by 15–60 min of uptake (Fig. 1*D*). Thus, total time of exposition to hypotonicity was 20 min in the shorter groups and 90 min in the longer groups. As shown in Fig. 1*D*, when using a shorter period of hypotonic incubation, the Cl^−^-dependent ^86^Rb^+^ uptake was similar between wild type and double mutant KCC3-T991A/T1048A injected oocytes (0.45 ± 0.25 *versus* 0.54 ± 0.35 nmol/oocyte/h; *p* = not significant), whereas when using a longer period of exposition to hypotonic medium, the Cl^−^-dependent, ^86^Rb^+^ uptake induced by wild type was significantly higher than the double mutant.

##### SPAK Kinase Phosphorylates KCC3 at Ser-96

**FIGURE 2.**
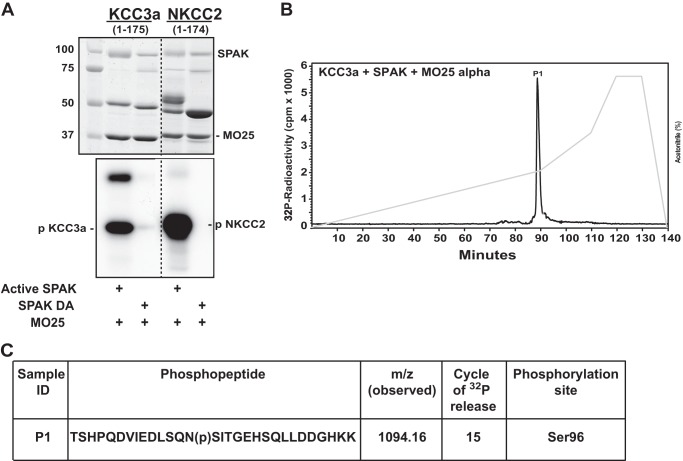
***In vitro* identification of KCC3 phosphorylation sites by SPAK.** *A*, GST-KCC3(1–175) and GST-NKCC2(1–174) were expressed in *E. coli* and phosphorylated with the active and kinase inactive (*DA*) forms of SPAK in the presence of 10 m MO25α. *Dotted lines* between autoradiographs and gels indicate that these were undertaken on separate gels. *p*, phosphorylated. *B*, phosphorylated GST-KCC3(1–175) was digested with trypsin and chromatographed on a C18 column. The peak fraction containing the major ^32^P-labeled peptides is labeled *P1. C*, summary of the mass spectrometry and solid-phase Edman sequencing data obtained after phospho-peptide analysis.

The evidence shows that phosphorylation and activation of the NKCCs in the N-terminal domain by SPAK and OSR1 mediate WNK kinase signaling (23–26). Therefore we decided to analyze the effect of SPAK on the phosphorylation of KCC3. To test this, a fragment of the cotransporter encompassing the N-terminal cytoplasmic domain, equivalent to the region that SPAK phosphorylates on NCC, NKCC1, and NKCC2 (10, 16), was phosphorylated in the presence of catalytically active recombinant SPAK complexed to its regulatory MO25 subunit (27). Strikingly, this revealed that active wild type but not kinase-inactive SPAK directly phosphorylated the N-terminal fragment of KCC3 encompassing residues 1–175 to a similar extent as the phosphorylation of the N-terminal domain of NKCC2 (Fig. 2*A*).

SPAK phosphorylated KCC3(1–175) in a time-dependent manner, showing an ∼18-fold increase in phosphorylation when compared with phosphorylation by active SPAK in the absence of MO25 (data not shown). ^32^P-labeled KCC3(1–175) was digested with trypsin and analyzed by chromatography on a C_18_ column for phospho-site mapping. One major ^32^P-labeled phospho-peptide was observed (Fig. 2*B*). A combination of solid-phase Edman sequencing and mass spectrometry (Fig. 2*C*) revealed that this encompassed a peptide phosphorylated at Ser-96. Mutating Ser-96 to Ala prevented phosphorylation of KCC3 by SPAK, thereby confirming that this residue represents the major site of phosphorylation (data not shown).

**FIGURE 3.**
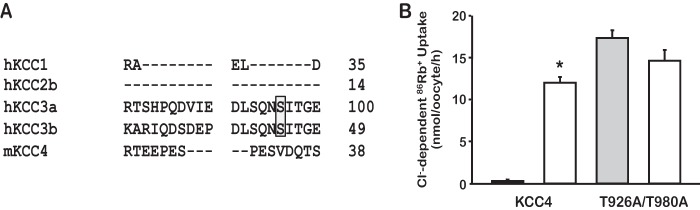
**Serine 96 is unique to KCC3.** *A*, KCCs sequence alignment of the N-terminal domain fragment revealed that Ser-96 of KCC3a (Ser-45 in KCC3b as shown the *gray shaded box*) is not present in other KCCs and thus is unique to KCC3. *h*, human; *m*, mouse. *B*, KCC4 Rb^+^ uptake of WT KCC4 and KCC4-T926A/T980A under isotonic (*gray bars*) and hypotonic (*open bars*) conditions. *n* = 8–9 experiments, *, *p* < 0.0001 *versus* KCC4 in isotonicity.

The sequence alignment of the four K^+^:Cl^−^ cotransporters shows that the potential phosphorylation site Ser-96 is unique to KCC3 because it is only present in KCC3a and KCC3b isoforms (Fig. 3*A*), but not in the other KCCs. The double mutant KCC4-T926A/T980A, which is homologous to KCC3-T991A/T1048A, but lacks the Ser-96 residue, is also active in isotonic conditions, but cannot be further increased in hypotonicity (Fig. 3*B*). Thus, in contrast to KCC3, in the KCC4 isoform that lacks the Ser-96 site, elimination of the two known sites at the C-terminal domain results in full activation of the cotransporter in isotonicity, suggesting that the presence of Ser-96 in KCC3 is responsible for the different functional behavior between KCC3-T991A/T1048A and KCC4-T926A/T980A toward hypotonicity.

##### KCC3 Serine 96 Becomes Dephosphorylated during Cotransporter Activation by Hypotonicity

**FIGURE 4.**
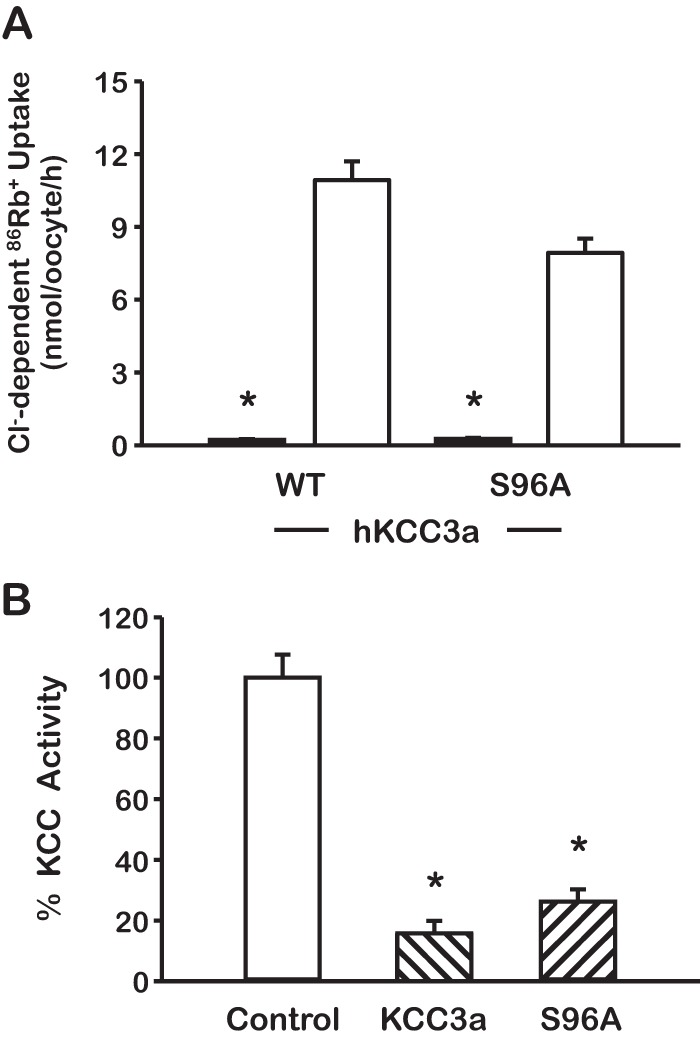
**Elimination of serine 96 by itself had no effect on the KCC3 behavior.** *A*, functional expression assay of WT KCC3a and mutant KCC3a-S96A shows no difference in hypotonic activation (*open bars*), with no basal expression under isotonic conditions (*gray bars*). *n* = 4, *p* < 0.0001 *versus* hypotonicity. *B*, similarly to WT KCC3a, the activity of mutant KCC3a S96A in hypotonic conditions is inhibited by WNK3 (*hatched bars*). *n* = 4, *, *p* < 0.0001 *versus* control.

We next substituted the serine 96 on wild type KCC3 to produce the KCC3-S96A mutant. As shown in Fig. 4*A*, similar to wild type KCC3, the KCC3-S96A was inactive in isotonic conditions and was dramatically stimulated by incubation in hypotonicity. The activation was fully prevented by co-expression with WNK3 (Fig. 4*B*). Thus, mutation of Ser-96 by itself has no effect on the behavior of KCC3 toward isotonic or hypotonic conditions, as well as to the known inhibitory action of WNK3 on the cotransporter (15, 18, 19).

**FIGURE 5.**
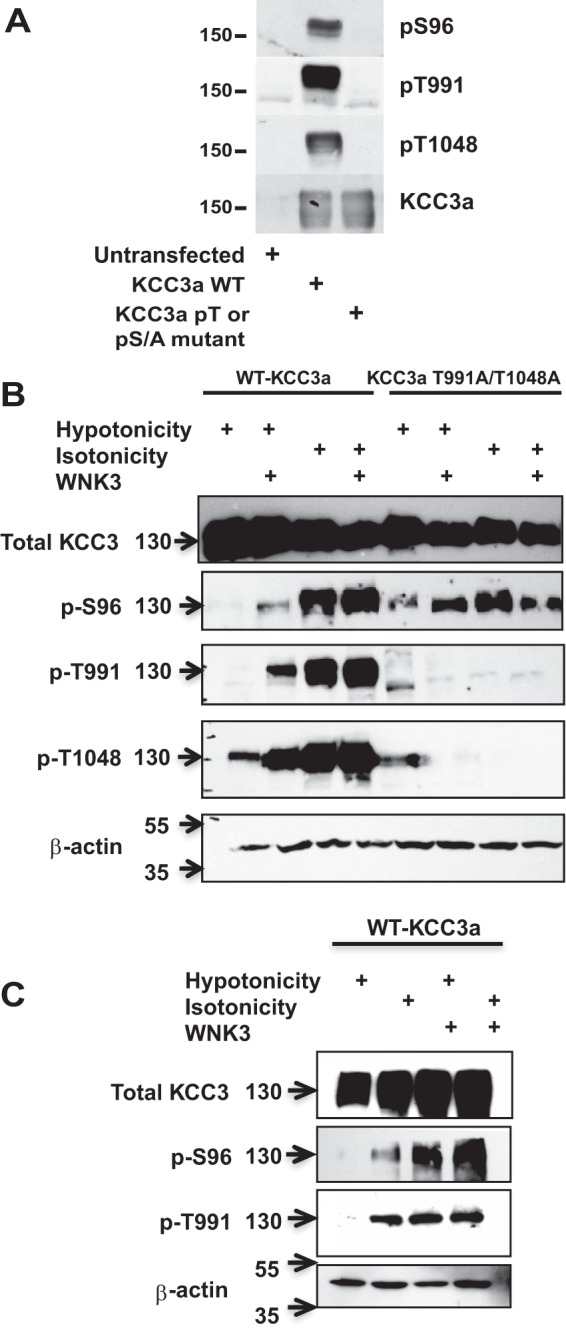
**Serine 96 becomes phosphorylated under isotonic conditions and in co-expression with WNK3.** *A*, characterization of KCC3a phosphorylation sites using phospho-specific antibodies. HEK 293 cells were transfected with WT human KCC3a or the indicated mutant forms (*pT* or *pS*). At 36 h after transfection, cells were lysed, and total cell extracts were immunoblotted with KCC3a total and phospho-specific antibodies. Similar results were obtained in 2 separate experiments. *B*, representative immunoblotting of total protein extracted from oocytes injected with WT KCC3a or KCC3a-T991A/T1048A cRNA with or without WNK3 cRNA and under hypotonicity or isotonicity maneuvers, as stated. *C*, representative immunoblotting of total proteins extracted from HEK293 cells transfected as stated. Blots were performed using specific antibody against total KCC3 and phospho-antibodies directed to Ser-96, Thr-991, and/or Thr-1048 of KCC3a and β-actin as loading control.

We next generated phospho-specific antibodies that efficiently recognize KCC3 phosphorylated at Ser-96, Thr-991, or Thr-1048. Mutation of the phosphorylated residue to a nonphosphorylatable Ala residue prevented recognition, confirming the specificity of the phospho-specific antibodies (Fig. 5*A*). As shown in Fig. 5*B*, we were able to demonstrate that wild type KCC3 overexpressed in *X. laevis* oocytes is indeed phosphorylated at these three residues in isotonic conditions, when the cotransporter is inactive, and is considerably dephosphorylated following incubation in hypotonic conditions, when KCC3 becomes active. We also observed the expected effect of WNK3 kinase co expression; in the presence of WNK3, dephosphorylation of the three sites was partially precluded. This correlates with the uncompleted activation of KCC3 in hypotonic conditions that occurs in the presence of this kinase (15, 18, 19). Similarly, in the double mutant KCC3-T991A/T1048A, Ser-96 also appeared phosphorylated in isotonic conditions and dephosphorylated after hypotonicity, and this was partially prevented by WNK3. These observations were corroborated using HEK293 cells transiently transfected with KCC3 cDNA and HEK293 cells stably transfected with WNK3 cDNA that were, in addition transiently transfected with KCC3 cDNA (19). As shown in Fig. 5*C*, HEK293 cells expressed KCC3 protein after transfection. In isotonic conditions, both Ser-96 and Thr-991 were phosphorylated, and the signal for each phospho-antibody disappeared when cells where incubated in hypotonicity, in which KCC activity is known to be increased (19). In addition, in HEK293 cells stably transfected with WNK3, Ser-96 and Thr-991 were phosphorylated in both isotonic and hypotonic conditions, consistent with the known inhibition that WNK3 exerts on KCC3 activity (15, 18, 19). All these observations together suggested that although Ser-96 is not a unique or a master site for KCC3 regulation, it behaves as phospho-sites Thr-991 and Thr-1048 and it seems to be a third site playing a role in full activation/inhibition of the cotransporter.

##### Serine 96 Is a Third Site Involved in Regulation of KCC3 Activity

**FIGURE 6.**
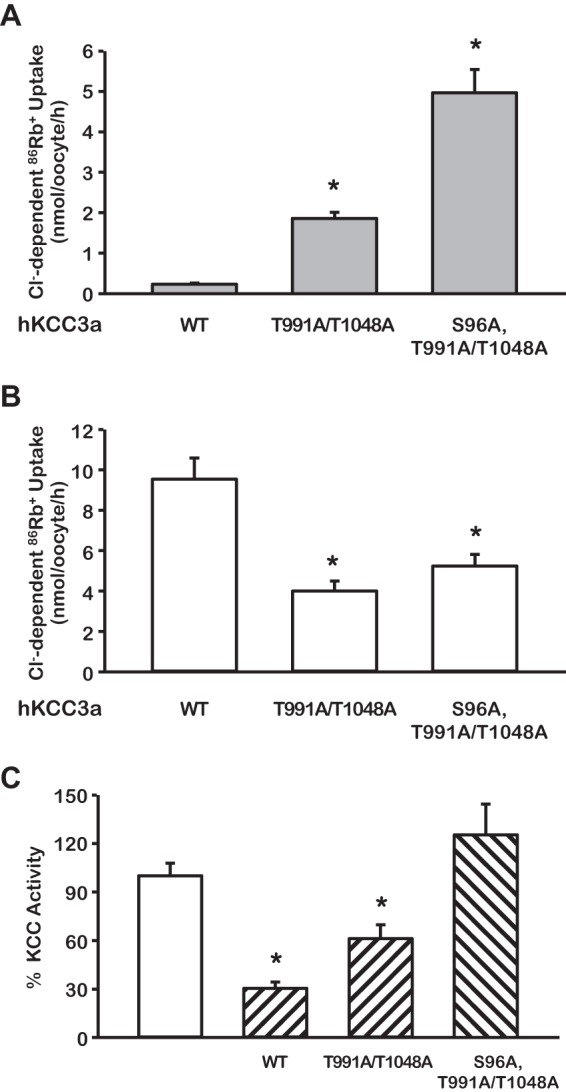
**Serine 96 is a third site involved in regulation of KCC3a activity.** *A* and *B*, functional expression assay was assessed in isotonic (*A*) or hypotonic (*B*) conditions in oocytes injected with cRNA for WT KCC3, KCC3 double mutant (T991A/T1048A), or KCC3 triple mutant (S96A/T991A/T1048A) as stated. *, *p* < 0.001 *versus* WT. *C*, the triple mutant KCC3a S96A/T991A/T1048A is no longer sensitive to WNK3 (*hatched bars*) inhibition. The *open bar* shows the control uptake for each clone taken as 100%. The *hatched bars* show the effect of WNK3 upon wild type or mutants KCC3, as stated. *n* = 3, *, *p* < 0.001 *versus white bar*.

Because Ser-96 residue in the double mutant KCC3-T991A/T1048A was phosphorylated and dephosphorylated similarly as in the wild type KCC3a, but elimination of this site alone had no effect on the cotransporter behavior (Fig. 4), to find out the role of serine 96 on the regulation of KCC3, we substituted the Ser-96 for alanine in the double mutant KCC3-T991A/T1048A to create the triple mutant KCC3-S96A/T991A/T1048A. In parallel experiments, functional expression of the double and triple mutant revealed that both were active in isotonic conditions (Fig. 6*A*). However, the level of activity was significantly higher in the triple mutant than in the double mutant (4.97 ± 0.57 *versus* 1.86 ± 0.15 nmol/oocyte/h; *p* < 0.0001), suggesting that the absence of phosphorylation in serine 96 contributed to higher activity of the triple mutant KCC3 in isotonic medium. This is supported by the findings observed during incubation of oocytes in hypotonic conditions. Further activation of the double mutant was noted (Figs. 1, *A* and *C*, and 6*B*), whereas no additional effect of hypotonicity was detected in the triple mutant (Fig. 6*B*). The Cl^−^-dependent, ^86^Rb^+^ uptake of the triple mutant in isotonicity and hypotonicity was 4.97 ± 0.57 and 5.23 ± 0.57 nmol/oocyte/h, respectively (*p* = not significant). In addition, co-injection of oocytes with WNK3 cRNA was able to significantly reduce the activity of wild type and double mutant KCC3 in hypotonic conditions, but no inhibitory effect was observed upon the triple mutant (Fig. 6*C*).

## DISCUSSION

In the present study, we provide functional and biochemical evidences suggesting that full activation by cell swelling of the K^+^:Cl^−^ cotransporter isoform KCC3 is only achieved after dephosphorylation of three distinct residues, two of which are located at the C-terminal domain (threonine residues 991 and 1048) and were reported previously by Rinehart *et al.* (11). The third residue, proposed in this study, is the serine 96 located at the N-terminal domain.

Our proposal for the requirement of a third site dephosphorylation to achieve complete activation of KCC3 differs from conclusions presented by Rinehart *et al.* (11). They observed, on the one hand, that double mutant KCC3-T991A/T1048A exhibited similar activity in both isotonicity and hypotonicity, and on the other hand, that activity of the double mutant in isotonicity was higher than that shown for wild type KCC3 when cells were incubated in hypotonic conditions. Thus, they suggested that Thr-991 and Thr-1048 were the only two residues required for cell swelling to induce KCC3 activation. However, although we observed significant activity of the double mutant KCC3-T991A/T1048A in isotonic conditions, we also documented that incubation of oocytes in hypotonicity further increased the activity of the double mutant KCC3-T991A/T1048A (Fig. 1*A*). The observed increase was sensitive to WNK3 (Fig. 1*C*). Because WNK3 cannot phosphorylate neither Thr-991 nor Thr-1048 in the double mutant KCC3-T991A/T1048A, this observation suggested that it was probably reducing the cotransporter activity by phosphorylating Ser-96. In addition, we showed that wild type KCC3 activity in hypotonic medium was significantly higher than that of the double mutant KCC3-T991A/T1048A in the same conditions (Fig. 1*A*). The reason for the discrepancy appears to be the time of exposure to the hypotonic medium. Rinehart *et al.* (11) used a total of 8 min, whereas we used longer exposure times. In this regard, it has been suggested that time of exposure to hypotonicity is associated with activation of the cotransporter (20–22). This is supported by our observations in Fig. 1*D* in which no difference between wild type and double mutant KCC3-T991A/T1048A was observed at a short period of incubation, whereas a significant difference was observed with longer exposure to hypotonic medium. Because the double mutant KCC3-T991A/T1048A is already active in isotonicity, then at a short period of incubation in hypotonicity, the activation of wild type KCC3 is not yet enough to reveal a difference. Thus, our functional observations suggested that at least a third phosphorylation site was involved in the regulation of KCC3 by extracellular osmolarity.

The potential phosphorylation site Ser-96 in KCC3 was detected by mass spectrometry. Because in the Na^+^ coupled-chloride cotransporters of the SLC12 family NCC, NKCC1, and NKCC2 regulatory phosphorylation sites have been detected at the N-terminal domain (28–31), and it has been demonstrated that SPAK is the kinase responsible for the phosphorylation of these sites (10, 16), we analyzed the effect of SPAK, complexed with its regulatory subunit MO25 (27), on the phosphorylation of the N-terminal domain of KCC3. NKCC2 N-terminal domain was used as positive control. Following this strategy, phosphorylation of Ser-96 in the KCC3a N-terminal domain was detected. Rinehart *et al.* (11) did not observe this site, probably due to methodological differences. On the one hand, what they did was to compare KCC3 phosphorylation status in isotonicity *versus* hypotonicity, whereas we directly applied SPAK/MO25 into the *in vitro* reaction. On the other hand, they analyzed KCC3 extracted from cells after 5 min of exposure to hypotonic conditions, which, as was discussed above, could not be enough time to allow a complete dephosphorylation of the cotransporter to detect a difference in this site.

Specific phospho-antibodies against Ser-96, Thr-991, and Thr-1048 were raised and used to demonstrate that indeed, in isotonic conditions in which KCC3 is inactive, all three sites were phosphorylated. Incubation of oocytes in hypotonicity resulted in decreased phosphorylation of the three sites, suggesting that dephosphorylation of these sites is coupled with activation of the cotransporter. Supporting this conclusion, when oocytes were co-injected with WNK3 cRNA, a kinase that is known to inhibit the KCCs (15), the activity and dephosphorylation of KCC3 in the three sites were partially precluded. Similar observations were obtained for Ser-96 and Thr-991 sites in HEK293 cells. Finally, the behavior of mutant S96A alone and in the context of the triple mutant S96A/T991A/T1048A also supports that Ser-96 is a third site involved in the modulation of the cotransporter activity. The absence of Ser-96 alone had no effect on KCC3 activity in isotonic conditions or its behavior toward activation by hypotonicity and inhibition by WNK3, suggesting that the C-terminal domain sites Thr-991 and Thr-1048 are hierarchically higher. However, elimination of Ser-96 in the absence of the other two sites resulted in a KCC3 that is more active than the double mutant in isotonic conditions and cannot be neither further activated by hypotonicity nor inhibited by WNK3, indicating that Ser-96 participates in the regulation of KCC3 activity.

The physiological relevance of a third site for regulation of the KCC3 cotransporter activity will require further investigation. This site is not present in other KCCs. Indeed, KCC4 in which both C-terminal domain threonine residues were eliminated (KCC4-T926A/T980A) resulted in full activation in isotonic conditions, suggesting that in this isoform no other sites are involved. Similarly, for NKCCs, between three and five different phosphorylation sites involved in their regulation have been located at the N-terminal domain, from which the master one is conserved (Thr-60 in human NCC), whereas some of the others are unique to one isoform (9, 24).

The presence of a third site suggests that KCC3 in some cells, tissues, or circumstances requires fine-tuning for its regulation. In this regard, it is worth noticing that we have shown before that an alternatively spliced isoform of KCC3a lacking exon 2 (KCC3a-x2M) is present in several human tissues (14). Exon 2 encodes for a 15-amino acid residue peptide in which Ser-96 is included. Thus, the major consequence of this splicing could be to produce KCC3 variants with and without Ser-96. Supporting the role of Ser-96 in the regulation of KCC3, we have reported previously that the speed of activation by hypotonicity of the KCC3a-x2M clone, lacking exon 2, is significantly faster than KCC3a containing exon 2 (14), suggesting that the absence of Ser-96 decreases the time required for full activation of the cotransporter because there is not a third site that has to be dephosphorylated. It will thus be interesting in future work to define which cells within a tissue are expressing variants with or without exon 2 or to raise knock-in mice of the different phosphorylation sites to explore the physiological consequence of the three different sites.

Phosphorylation/dephosphorylation processes modulate the activity of the SLC12 cotransporters. Several studies support that promoting phosphorylation by cell shrinkage, activating kinases, or inhibiting protein phosphatases increases the activity of the NKCCs and inhibits the KCCs, thus increasing intracellular chloride concentration, cell volume, and transcellular ion transport capacity. In contrast, promoting dephosphorylation by cell swelling, inhibiting kinases, or activating protein phosphatases reduces the NKCCs and activates the KCCs, thus decreasing intracellular chloride concentration, cell volume, and transcellular ion transport. Because the mode of operation of these secondary transporters is still far from being understood, it is not clear at the moment how the phosphorylation or dephosphorylation of one site can affect the transport activity of the protein, particularly because it is believed that this occurs with proteins that are already in the plasma membrane (9, 11, 32). Thus, affecting trafficking by the phospho-status of the protein does not seem to be the answer. One possibility could be that phosphorylating or not phosphorylating certain residues changes the structure of the protein, opening or closing pores or turning binding sites for ions more or less accessible, thus changing the transport capacity of the cotransporter. Another possibility is that phosphorylation on a given cotransporter could be modulating its ubiquitylation, thus indicating that phosphorylation/dephosphorylation processes also affect the amount of total cotransporter (33).

In summary, our data support that serine 96 in KCC3 is a third phosphorylation site implicated in the regulation of the cotransporter activity.

## Footnotes

4 The abbreviations used are:

KCC: K^+^:Cl^−^ cotransporter
N(K)CC: Na^+^-coupled chloride cotransporter
Bis-Tris: 2-(bis(2-hydroxyethyl)amino)-2-(hydroxymethyl)propane-1,3-diol
WNK: with no lysine kinase
SPAK: STE20/SPS1-related proline/alanine-rich kinase.
